# Nile Ward PICU violence reduction quality improvement project

**DOI:** 10.1192/bjo.2021.570

**Published:** 2021-06-18

**Authors:** Mehtab Rahman, Claudia Taylor, Roda Abdullahi, Anthony Okwuokei, Matthew Waugh, Mahomed Kaji, Biganani Magadlela

**Affiliations:** Central & North West London NHS Foundation Trust

## Abstract

**Aims:**

To reduce incidents of inpatient violence and aggression at Nile Ward Psychiatric Intensive Care Unit (PICU), St Charles Hospital by at least 30% between December 2019 and December 2020. Reducing inpatient violence is a major quality improvement (QI) priority for CNWL NHS Foundation Trust.

**Method:**

As a Psychiatric Intensive Care Unit, Nile Ward looks after male patients suffering from severe mental illness (SMI). This usually includes patients presenting with high levels of violent and aggressive behaviour. Prior to this QI project, there were high levels of patient assaults towards staff and other patients. This required a lot of medication use, including rapid tranquilisation, restraint and the use of seclusion. This QI project was started to allow the Nile MDT to explore ways to reduce serious incidents on the ward in the least restrictive manner.

We implemented a number of change ideas within this project. Our change ideas included: 1. A new risk management tool : ‘Ragging', a daily risk assessment tool, was created to assess patients’ risk of violence and aggression to allow signposting of appropriate interventions to safely manage risk. 2. A brand new Staff Photo board : New photos of all permanent and bank staff displayed in the ward with no hierarchy of positions. 3. A new Patient Feedback board : Patient experience, comments and feedback displayed in common areas of the ward which are regularly updated. 4. Mutual Expectations between Staff and Patients: A set of expectations created in co-production with patients displayed in the communal areas of the ward to be followed by both staff and patients. 5. Gardening sessions : One of our newer change ideas during the COVID-19 pandemic was to provide a safe, socially distanced space for patients to be involved in growing and caring for the Nile Ward garden with our activities co-ordinator. 6. Optimisation of Physical Exercise : Focus on physical activity through garden fitness sessions and 1-1 fitness sessions in the gym. This was another change idea commenced during the COVID-19 pandemic. These sessions occur throughout the day with our fitness instructor and enable our patients to focus on their physical health & fitness. 7. Improved Ward Environment : Gym equipment were upgraded and the appearance of the ward gymnasium was enhanced using quality art created in co-production with patients.

**Result:**

There was a 43% reduction in the number of violent incidents in the ward following QI interventions. The details of the results will be depicted in pictorial form in the poster.

**Conclusion:**

Our patients are able to recover in a safe environment with a reduced level of violence and aggression resulting in patients receiving less rapid tranquilisation and restrictive interventions. We have had fewer assaults on staff which has made our staff feel safer to work in a busy PICU. Staff feel more confident in their role through the use of the new risk assessment tool . Patients and staff alike have given positive feedback to the changes implemented in this QI project, with violence being successfully reduced by 43%. We hope that our QI project can be used as an example to show how QI methodology can enable Violence Reduction within mental health services.

